# Physiological roles of Arabidopsis MCA1 and MCA2 based on their dynamic expression patterns

**DOI:** 10.1007/s10265-024-01575-8

**Published:** 2024-08-28

**Authors:** Miki Kubota, Kendo Mori, Hidetoshi Iida

**Affiliations:** 1 Department of Biology, Tokyo Gakugei University, 4-1-1 Nukuikita-Machi, Koganei, Tokyo 184-8501 Japan; 2 Kawagoe Minami High School, 1-21-1 Minamiotsuka, Kawagoe, Saitama 350-1162 Japan; 3 Tamagawa Academy High School, 2713 Naracho, Aoba Ward, Yokohama, Kanagawa 227-0036 Japan

**Keywords:** *Arabidopsis thaliana*, Ca^2+^-permeable, Gene expression, GUS staining, MCA, Mechanosensitive channel, Plant development

## Abstract

Determining the mechanisms by which plants sense and respond to mechanical stimuli is crucial for unraveling the detailed processes by which plants grow and develop. Mechanosensitive (MS) channels, including MCA1 and its paralog MCA2 in *Arabidopsis thaliana*, may be essential for these processes. Although significant progress has been made in elucidating the physiological roles of MS channels, comprehensive insights into their expression dynamics remain elusive. Here, we summarize recent advancements and new data on the spatiotemporal expression patterns of the *MCA1* and *MCA2* genes, revealing their involvement in various developmental processes. Then, we describe findings from our study, in which the expression profiles of *MCA1* and *MCA2* were characterized in different plant organs at various developmental stages through histochemical analyses and semiquantitative RT‒PCR. Our findings revealed that *MCA1* and *MCA2* are preferentially expressed in young tissues, suggesting their pivotal roles in processes such as cell division, expansion, and mechanosensing. Lastly, we discuss the differential expression patterns observed in reproductive organs and trichomes, hinting at their specialized functions in response to mechanical cues. Overall, this review provides valuable insights into the dynamic expression patterns of *MCA1* and *MCA2*, paving the way for future research on the precise roles of these genes *in planta*.

**Supplementary Information:**

The online version contains supplementary material available at 10.1007/s10265-024-01575-8.

## Introduction

Mechanical forces profoundly influence plant growth and development, necessitating sophisticated mechanisms for sensing and responding to stimuli; these stimuli are produced by environmental forces and intrinsic force-generation events, such as cell division and expansion. (Hamant et al. 2008; Louveaux et al. 2016). Among these mechanisms, mechanosensitive (MS) channels have emerged as crucial components that help transduce mechanical signals into biochemical responses. In *Arabidopsis thaliana* (L.) Heynh. (Arabidopsis), MCA1 (*mid1*-complementing activity (1) and its paralog MCA2, which belong to the MS channel family, have garnered significant attention due to their roles in mediating various physiological processes (Hamilton et al. 2015; Kurusu et al. 2013). While previous studies have provided valuable insights into their functions, comprehensive knowledge on their expression dynamics is still needed to decipher their precise roles *in planta*. In this review, we explore the potential functions of MCA1 and MCA2 in Arabidopsis, emphasizing recent progress in elucidating the spatiotemporal expression profiles of *MCA1*p::*GUS*, the *MCA1* promoter fused to the reporter gene β-glucuronidase (*GUS*), and *MCA2*p::*GUS*. Through this review, we aim to illuminate the possible physiological roles of MCA1 and MCA2 in various developmental processes.

## Overview of MCA channels

The MCA1 and MCA2 proteins in Arabidopsis consist of 521 and 516 amino acid residues, respectively, and share 73% amino acid sequence identity. Both proteins exhibit identical structural features, including a single transmembrane segment near the N-terminus, an EF-hand-like motif slightly beyond the middle region on the N-terminal side, a coiled-coil motif in the middle, and a PLAC8 motif in the C-terminal half (Nakagawa et al. 2007). These proteins also form a homotetramer to produce Ca^2+^-permeable channels in yeast (Nakano et al. 2011), insect cells (Shigematsu et al. 2014) and in vitro (Yoshimura et al. 2021).

Although Arabidopsis MCA1 and MCA2 are not always studied comparatively, certain research has shown that the proteins share some physiological roles and also have differences. The growth of the *mca1*-null *mca2*-null double mutant, but not that of single mutants, is sensitive to excess external Mg^2+^; therefore, the physiological roles of MCA1 and MCA2 for plant growth may overlap under excess Mg^2+^ conditions (Yamanaka et al. 2010). MCA1 and MCA2 are involved in the response to hypergravity in hypocotyls (Hattori et al. 2020). Both mediate a cold-induced increase in the cytosolic free calcium concentration, [Ca^2+^]_cyt_. (Mori et al. 2018). MCA1, but not MCA2, is needed for primary roots to enter hard agar media from soft agar media (Nakagawa et al. 2007; Yamanaka et al. 2010). Moreover, MCA2, but not MCA1, plays a role in Ca^2+^ uptake in roots (Nakayama et al. 2010). Researchers have shown that MCA1 is involved in the hypoosmotic shock-induced increase in [Ca^2+^]_cyt_ in seedlings (Nakagawa et al. 2007) and in the gravity-induced increase in [Ca^2+^]_cyt_ (Nakano et al. 2021). An electrophysiological study with a *Xenopus* oocyte system suggested that MCA1 and MCA 2 are MS channels (Furuichi et al. 2012). A recent in vitro electrophysiological study clearly demonstrated that MCA1 and MCA2 are MS channels that are inherently sensitive to membrane stretching, a type of mechanical force (Yoshimura et al. 2021).

In some cases, MCA1 and MCA2 perform different functions because the location of their expression is different. For example, as mentioned above, MCA1, but not MCA2, is necessary for primary roots to enter hard agar medium from soft agar medium (Nakagawa et al. 2007; Yamanaka et al. 2010). This difference may be explained by the differential expression of MCA1 and MCA2 in the root tip (Yamanaka et al. 2010). Unlike *MCA2*p::*GUS*, *MCA1*p::*GUS* was shown to be expressed at the Arabidopsis primary root tip (Yamanaka et al. 2010), suggesting that MCA1 is responsible for detecting the hardness of agar.

This is a good example of how the physiological roles of MS channels can be well linked with their localization. Such known examples are as follows: The Arabidopis pollen-specific aquaporins, NIP4;1 and NIP4;2, are required for pollen development and pollinations through water transport (Giorgio et al. 2016), while one of the highest expressed PIP1 members in leaves, AtPIP1;2, acts as a CO_2_ transport facilitator (Heckwelf et al. 2011; Weig et al. 1997). The root localization of the Arabidopsis nitrate transporter NRT2.1 is involved in nitrate uptake from the soil, while the seed localization of NRT2.7 is responsible for nitrate storage into the seed (Xu et al. 2024).

## Expression patterns in leaves

The expression patterns of MCA1 and MCA2 in leaves are intriguing, suggesting that these proteins play crucial roles in leaf development. Our histochemical analyses with standard methods (see Table S1) revealed that both genes were preferentially expressed in younger cotyledons and leaves.

### Cotyledons

**Fig. 1:**
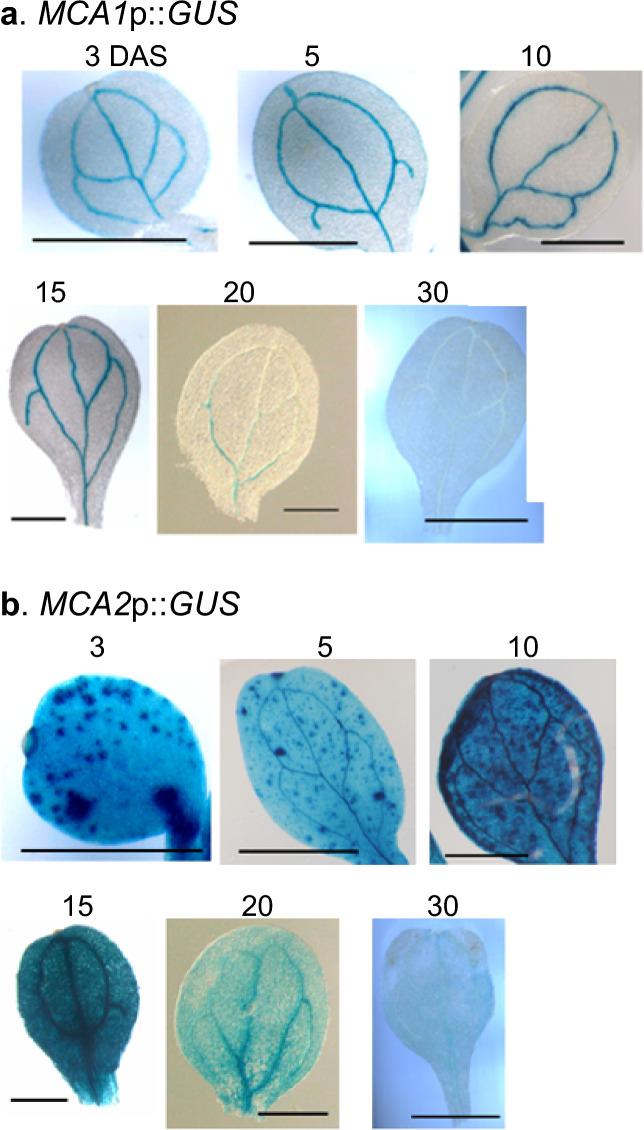
Developmental changes over time in the expression patterns of *MCA1*p::*GUS* and *MCA2*p::*GUS* in cotyledons. Cotyledons of *MCA1*p::*GUS* plants and *MCA2*p::GUS plants at 3, 5, 10, 15, 20 and 30 DAS were GUS-stained and photographed using a binocular stereomicroscope. This figure shows a typical example from three or more experiments. Similar results were seen in the *MCA1*p::*GUS* lines 1, 3, and 5, and the *MCA2*p::*GUS* lines 3, 4, and 5. Therefore, we examined at least nine samples independently. Scale bar = 0.1 cm

Previous studies have reported that both *MCA1*p::*GUS and MCA2*p::*GUS* are expressed in the cotyledons of 10-day-old Arabidopsis plants (Yamanaka et al. 2010). However, the analysis is limited to 10-day-old plants, and detailed studies, including those on spatiotemporal expression, have not been conducted at all. Therefore, we conducted the analysis of their spatiotemporal expression patterns. Figure 1a shows that *MCA1*p::*GUS* is stably expressed in the veins of cotyledons up to 15 days after sowing (DAS), but its expression decreases at 20 DAS and becomes undetectable at 30 DAS. Figure 1b shows that *MCA2*p::*GUS* was expressed throughout the veins and mesophyll cells of the cotyledons up to 15 DAS, with scattered points indicating particularly high expression; however, at 20 and 30 DAS, the expression in both the veins and mesophyll cells was greatly reduced (Fig. 1b). The above results suggest that the expression of both *MCA1* and *MCA2* is high in young cotyledons and gradually decreases as cotyledons age.

### True leaves

**Fig. 2:**
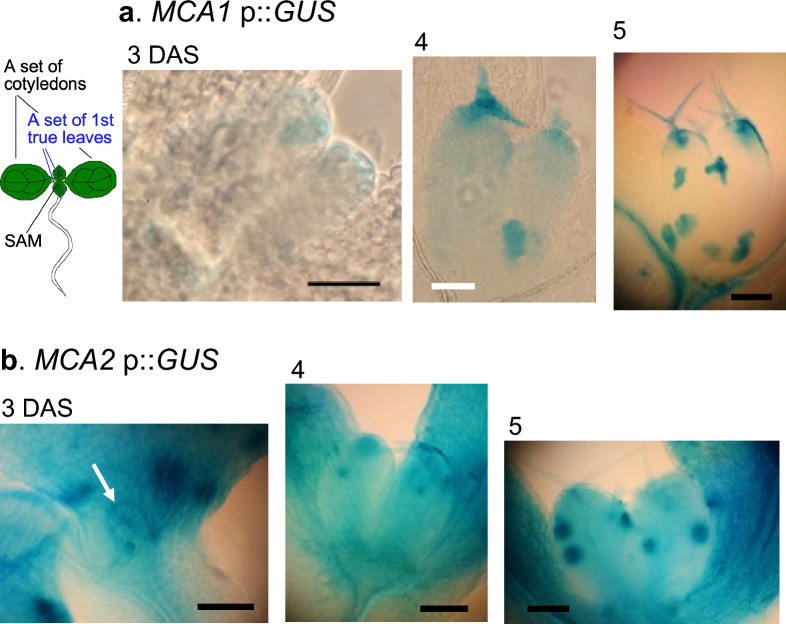
Expression patterns of *MCA1*p::*GUS* and *MCA2*p::*GUS* in the leaf primordia. Seedlings including the shoot apical meristem (SAM) of *MCA1*p::*GUS* plants and *MCA2*p::GUS plants at 3, 4, and 5 DAS were GUS-stained and photographed using a binocular stereomicroscope. This figure shows a typical example from three or more experiments. Similar results were seen in the *MCA1*p::*GUS* lines 1, 3, and 5, and the *MCA2*p::*GUS* lines 3, 4, and 5. Therefore, we examined at least nine samples independently. The white arrow points to the top of the leaf primordium. Scale bar = 50 μm

Leaf development starts at the shoot apical meristem (SAM), from which the leaf primordium emerges (Barton 2010). In an investigation on the expression of *MCA1* and MCA2 in leaf primordia, histochemical analysis of the leaf primordia was performed at 3, 4, and 5 DAS. As shown in Fig. 2a, histochemical analysis revealed that the expression of *MCA1*p::*GUS* was weak and restricted to the tip of the leaf primordium. At 5 DAS, patchy expression and tip expression were detected. In contrast, the expression of *MCA2*p::*GUS* was observed throughout the entire leaf primordium, although it was more pronounced at the tips (Fig. 2b). At 5 DAS, patchy expression was also observed. These results suggest that MCA1 and MCA2 are involved in early development of the SAM, albeit to varying degrees.

**Fig. 3:**
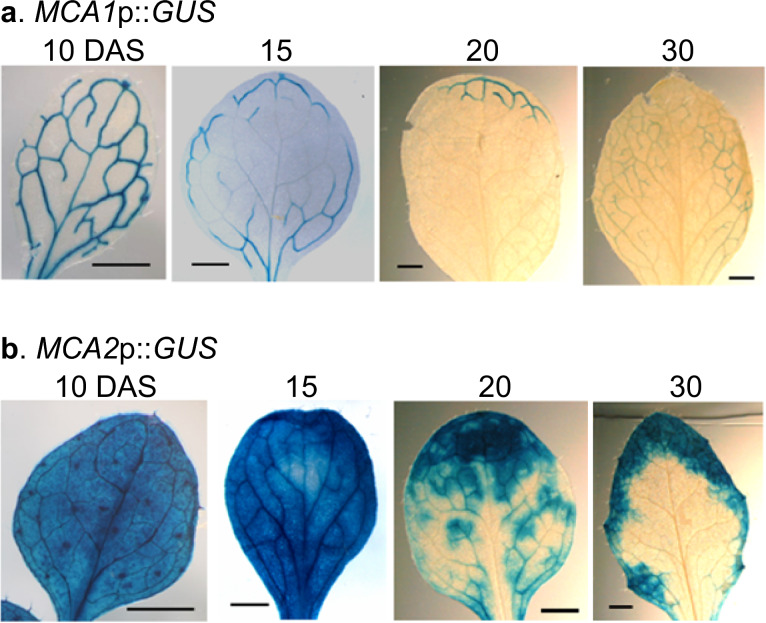
Developmental changes in the expression patterns of *MCA1* and *MCA2* in the first true leaves. The first true leaves of *MCA1*p::*GUS* plants and *MCA2*p::*GUS* plants at 10, 15, 20 and 30 DAS were GUS-stained and photographed using a binocular stereomicroscope. This figure shows a typical example from three or more experiments. Similar results were seen in the *MCA1*p::*GUS* lines 1, 3, and 5, and the *MCA2*p::*GUS* lines 3, 4, and 5. Therefore, we examined at least nine samples independently. Scale bar = 0.1 cm

Researchers have shown that for the first set of true leaves, *MCA1*p::*GUS* is clearly expressed in the veins of these leaves at 10 DAS (Yamanaka et al. 2010), but its expression gradually decreases by 15 DAS (Fig. 3a). In this case, the expression tended to decrease mainly from the center of the first set of true leaves. At 20 and 30 DAS, very little *MCA1*p::*GUS* expression was observed.

*MCA2*p::*GUS* is expressed in veins and mesophyll cells at 10 through 30 DAS (Yamanaka et al. 2010; Fig. 3b), but its expression level is greater at 10 and 15 DAS than at 20 and 30 DAS (Fig. 3b). Interestingly, the expression levels tended to remain high at the tip of the first set of true leaves and decreased in the remainder of the leaves. This tendency is similar to that of *MCA1*p::*GUS*.

**Fig. 4:**
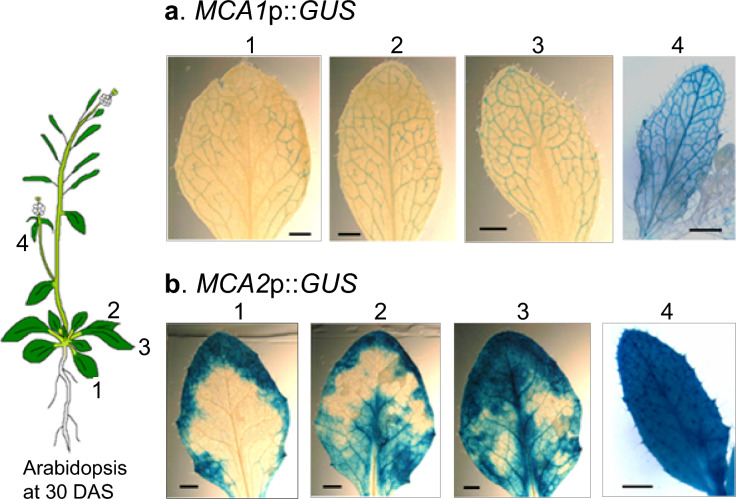
Expression patterns of *MCA1* and *MCA2* in the true leaves of plants at 30 DAS. True leaves of *MCA1*p::*GUS* and *MCA2*p::*GUS* plants at 30 DAS were GUS-stained and photographed using a binocular stereomicroscope. Note that the numbers 1 through 4 are the order of appearance of the youngest leaves; the lower the number, the older the leaf. This figure shows a typical example from three or more experiments. Similar results were seen in the *MCA1*p::*GUS* lines 1, 3, and 5, and the *MCA2*p::*GUS* lines 3, 4, and 5. Therefore, we examined at least nine samples independently. Note that the panels **a**1 and **b**1 of this figure are identical to those **a**30 and **b**30 of Fig. 3. Scale bar = 0.1 cm

Young leaves emerge as the plant body ages. It is interesting to examine whether *MCAs* expression is greatly influenced by the leaf age or the age of the plant body relative to the leaf. To test this possibility, we performed histochemical analysis on four leaves of different ages from plants at 30 DAS (Fig. 4). *MCA1*p::*GUS* expression was reduced in the veins of older (rosette) leaves (Fig. 4a, leaf No. 1–3), whereas it was clearly expressed in the veins of younger (cauline) leaves (Fig. 4a, leaf No. 4). In addition, *MCA2*p::*GUS* expression was lower in the veins and mesophyll cells of the central and surrounding areas of older (rosette) leaves(Fig. 4b, leaf No. 1–3), whereas *MCA2*p::*GUS* was widely and strongly expressed in the veins and mesophyll cells of younger (cauline) leaves (Fig. 4b, leaf No. 4). Therefore, *MCA1* and *MCA2* were found to be highly expressed in new leaves, suggesting that both gene products are functional in areas with active cell division and expansion during Arabidopsis development.

Similarly, a rice homolog and a maize homolog of Arabidopsis MCAs reportedly participate in cell division and expansion, which may contribute to the growth of plant organs and bodies (Kurusu et al. 2012; Liang et al. 2020; Liu et al. 2015; Rosa et al. 2017). These results suggest that MCA1 and MCA2 are involved in early development of the leaf, although it is likely that MCA1 is less involved in this event than MCA2 and spatially limited because the expression of MCA1 is less than that of MCA2 and limited to the tip of the young leaf. Since the expression of MCA1 and MCA2 is influenced by the leaf age, not the age of the plant body, the MCA family might be involved in regulating cell division and expansion in young leaves across species.

Cell division and expansion occur actively in young leaves, putting pressure on neighboring cells and tissues and leading to mechanical stress within the leaf structure. In addition, Ca^2+^ regulates cell division and expansion by modulating the activity of enzymes involved in cell wall synthesis and rigidity during leaf growth (Hepler 2005). Since *MCA1* and *MCA2* are expressed at the site where mechanical stress is added, it is likely that both gene products are involved in the leaf development because of their ability to sense and respond to mechanical stress as Ca^2+^-permeable channels (Furuichi et al. 2014; Nakagawa et al. 2007; Yoshimura et al. 2021). However, further experiments are needed to definitively establish this association.

## Spatiotemporal expression patterns in primary roots

**Fig. 5:**
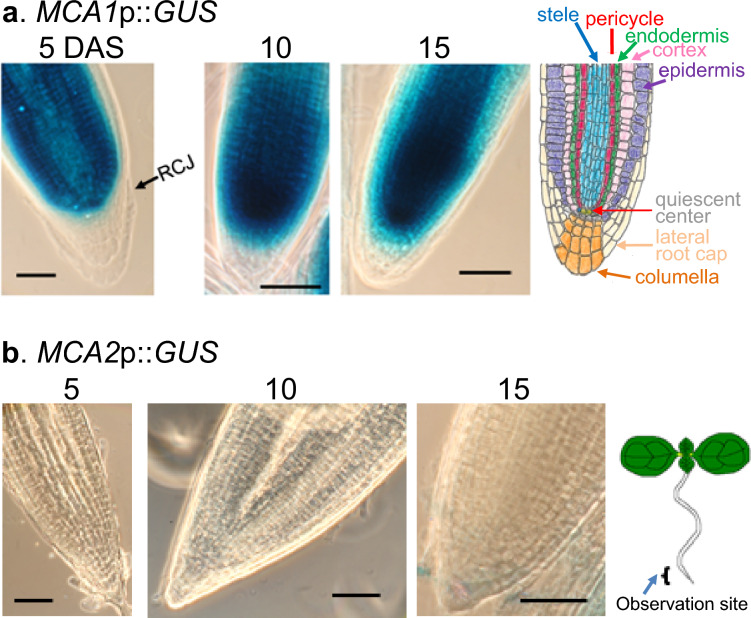
There were no detectable spatiotemporal changes in *MCA1* and *MCA2* expression patterns in the root meristematic zone over time. Primary roots, including the meristematic zone, of *MCA1*p::*GUS* and *MCA2*p::*GUS* plants at 5, 10 and 15 DAS were GUS-stained and photographed using a phase-contrast microscope. Naming of the meristematic zone was based on Verbelen et al. (2006), who defined that the meristem stretched up to 200 μm away from the root cap junction (RCJ). Note that only roots with clearly identifiable RCJs are marked. This figure shows a typical example from three or more experiments. Similar results were seen in the *MCA1*p::*GUS* lines 1, 3, and 5, and the *MCA2*p::*GUS* lines 3, 4, and 5. Therefore, we examined at least nine samples independently. Scale bar = 50 μm. The drawing of the root tip was hand-drawn with reference to the figures presented in three papers (Dolan et al. 1993; Rahni and Birnbaum 2019; Schres et al. 2002)

We found that the spatiotemporal expression patterns of *MCA1*p::*GUS* and *MCA2*p::*GUS* in primary roots remained unchanged for at least 15 DAS (Fig. 5). During the developmental period, *MCA1*p::*GUS* was stably expressed in the root tip areas, including the meristematic zone and the stele, pericycle, and endodermis of the transition and elongation zones; however, expression was not observed in the root cap, which included columellar cells (Yamanaka et al.^2010^). In contrast, *MCA2*p::*GUS* was not detected in the root tip. We cannot definitively state whether *MCA1*p::*GUS* is expressed in the cortex that generates faint GUS staining because the histochemical GUS assay accompanies the intercellular diffusion of reaction products (Guivarc’h et al. 1996). A cross-sectional experiment of primary roots has revealed that *MCA1*p::*GUS* is not expressed in the cortex (Yamanaka et al. 2010).

Although little is known about the sensitivity of the root meristematic zone to mechanical forces, this zone is involved in the response to external forces (Chiatante et al. 2021; Potocka et al. 2011). In addition, the cell division rate of the stele, in which *MCA1*p::*GUS* staining was the highest, was reported to be greater than that of surrounding tissues, such as the cortex and epidermis (Rahni and Birnbaum 2019). While the stele of the root apical meristem is exposed to mechanical stresses generated by both external and internal forces, it is important to consider that other regions of the root may also experience significant mechanical stresses. Additionally, the role of MCA1 in sensing mechanical stresses in the meristematic zone is not yet fully understood, and there may be other factors or mechanisms involved in this process. Therefore, It is plausible to assume that MCA1 could play a role in sensing mechanical stresses in the meristematic zone because of the expression of *MCA1p*::*GUS* (and not that of *MCA2*p::*GUS*), and the inability of the *mca1*-null mutant to penetrate harder agar medium. (Nakagawa et al. 2007; Yamanaka et al. 2010).

## Semiquantitative analysis of organ-specific expression

The histochemical analysis revealed the developmental patterns of *MCA1*p::*GUS* and *MCA2*p::*GUS* in plant organs. To confirm the above results using a different method, we employed semiquantitative reverse transcription‒polymerase chain reaction (RT‒PCR). For these experiments, we prepared total RNA from cotyledons, the first set of true leaves, the second set of true leaves, and primary roots of plants at 10, 15, and 20 DAS. Then, we performed a semiquantitative RT‒PCR analysis with the prepared total RNAs as templates to examine the organ-specific expression of *MCA1* and *MCA2*. mRNA encoding Arabidopsis tubulin β-1 (*TUB1* gene product) was used as an internal control to normalize the gene expression levels of the different samples.

**Fig. 6:**
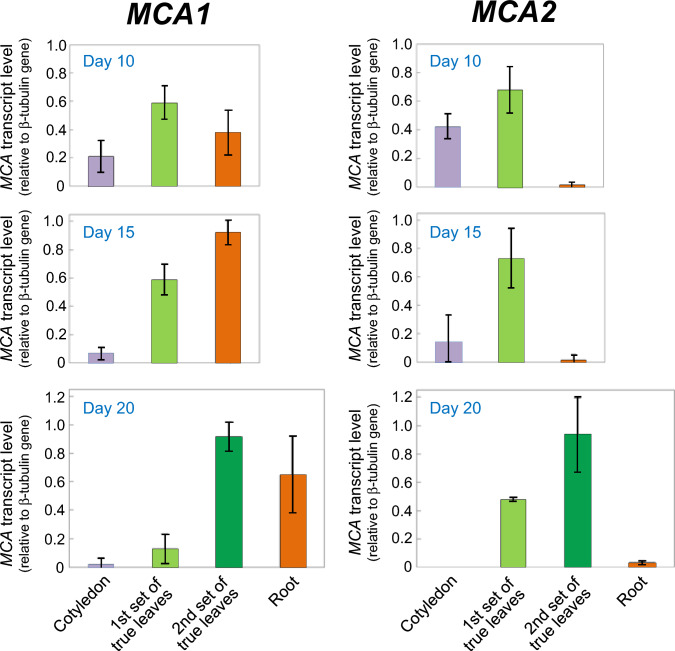
Developmental changes in transcript levels of *MCA1* and *MCA*2 in plants at 10, 15 and 20 DAS. Cotyledons, a 1st set of true leaves, a 2nd set of true leaves, and primary roots of approximately 10 wild-type plants at 10, 15, and 20 DAS were collected separately and frozen in liquid nitrogen. Total RNA was extracted from each plant, and cDNA was synthesized using the RNA as a template. The amount of *MCA1* and *MCA2* in the template mRNA was then detected by RT-PCR. The density of the amplified product bands was quantified by ImageJ and normalized to β-tubulin expression. The mRNA amounts of *MCA1* and *MCA2* per organ were compared as arbitrary units. This figure shows the value of *MCA1* or *MCA2* when the value of β-tubulin was set to 1. The values are the means of three independent experiments. Error bars indicate standard deviation

In cotyledons, the *MCA1* and *MCA2* levels were the highest in plants at 10 DAS and decreased at 15 DAS and further at 20 DAS (Fig. 6). In the first set of true leaves, the *MCA1* and *MCA2* levels were high in the plants at 10 and 15 DAS but decreased at 20 DAS. These results suggest that *MCA1* and *MCA2* are highly expressed when cotyledons and leaves are young. This finding was confirmed by the observation of the second set of true leaves that were the youngest and presented the highest expression levels. In primary roots, high expression levels of *MCA1* appeared to be maintained from 10 to 20 DAS, while *MCA2*p::*GUS* expression levels remained very low during the same developmental stage.

To summarize the expression data described above, the results obtained by semiquantitative RT‒PCR were consistent with those obtained by histochemical analyses; therefore, these data clearly show that the expression of *MCA1* and *MCA2* is the highest in newly produced organs.

## Expression patterns in the vasculature and lateral roots

**Fig. 7:**
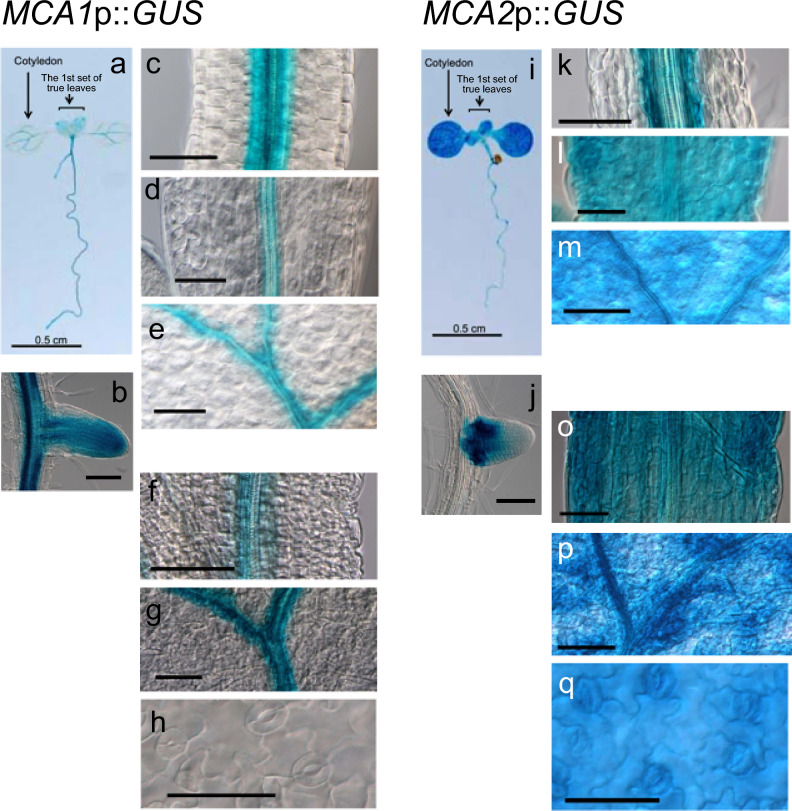
Expression patterns of *MCA1*p::*GUS* and *MCA2*::*GUS* in various plant parts. Tissue-specific expression of *MCA1*p::*GUS* and *MCA2*::*GUS* was investigated by differential interference microscopy. **a** and **i**, Full-body photo of a seedling at 7 DAS; **b** and** j**, lateral root primordia at 10 DAS; **c** and** k**, hypocotyl at 7 DAS;** d** and** l**, petiole of a cotyledon at 7 DAS; **e** and** m**, vein of a cotyledon at 7 DAS; **f** and** o**, petiole of the 1st true leaf at 10 DAS; **g** and** p**, vein of the 1st true leaf at 10 DAS; **h** and **q**, stomata. This figure shows a typical example from five experiments. Similar results were seen in the *MCA1*p::*GUS* lines 1, 3, 5, and 9, and the *MCA2*p::*GUS* lines 3, 4, and 5. Therefore, we examined at least 15 samples independently. Scale bar in **b**-**h** and **j**-**q** = 100 μm. Note that the magnified images (panels **b**-**g**, and **j**-**q**) are not simply a portion of panels a and i, respectively, but also a portion of other plant samples

We histochemically investigated the expression patterns of *MCA1*p::*GUS* and *MCA2*p::*GUS* in primary roots and leaves, although basic histochemical analysis has already been performed (Yamanaka et al. 2010). The vasculature, particularly the phloem, transmits mechanical stress-induced, long-distance Ca^2+^ signals in Arabidopsis (Toyota et al. 2018). Vascular tissues in plants play a role in the transportation of water, minerals, and nutrients, and Ca^2+^ has crucial roles in cell wall stability, signaling, and the regulation of enzyme activity (Thor 2019). Therefore, MCA1 and MCA2 expressed in this tissue may be involved in regulating the above events. Indeed, as previously shown, *MCA1*p::*GUS* was expressed in the vascular tissues of the cotyledons, true leaves, and primary roots (Fig. 7a-g). In addition, its expression in the primary root was undetectable in other specific cells or regions, including columellar cells, the lateral root cap, the epidermis, and the cortex (Fig. 5a). *MCA1*p::*GUS* was also expressed in the lateral root primordium (Fig. 7b), the stele of the hypocotyl (Fig. 7c), the vascular bundle of the petiole and vein of the cotyledon (Fig. 7d, e), and the petiole and vein of the first leaf (Fig. 7f, g). No expression of *MCA1*p::*GUS* was detected in stomatal guard cells (Fig. 7h) whose opening is controlled by turgor pressure, a mechanical force (Yi et al. 2022).

The expression patterns of *MCA2*p::*GUS* were different from those of *MCA1*p::*GUS*, although both were expressed in the stele and vascular bundles in addition to the surrounding tissues (Fig. 7k-p). *MCA2*p::*GUS* expression was observed in stomatal guard cells (Fig. 7q) but not the root tip (Fig. 5b). *MCA2*p::*GUS* was expressed at the base of the lateral root primordium but not at the distal end (Fig. 7j).

Additionally, *MCA1* and *MCA2* were strongly expressed in young lateral roots (Fig. 7b, j). The development of lateral root primordium encounters mechanical stress caused by the overlaying tissues (Pond 1908) and external factors, including soil compaction. Thus, it is plausible to assume that MCA1 and MCA2 are ivolved in the development of lateral roots.

## Expression patterns in reproductive organs

**Fig. 8:**
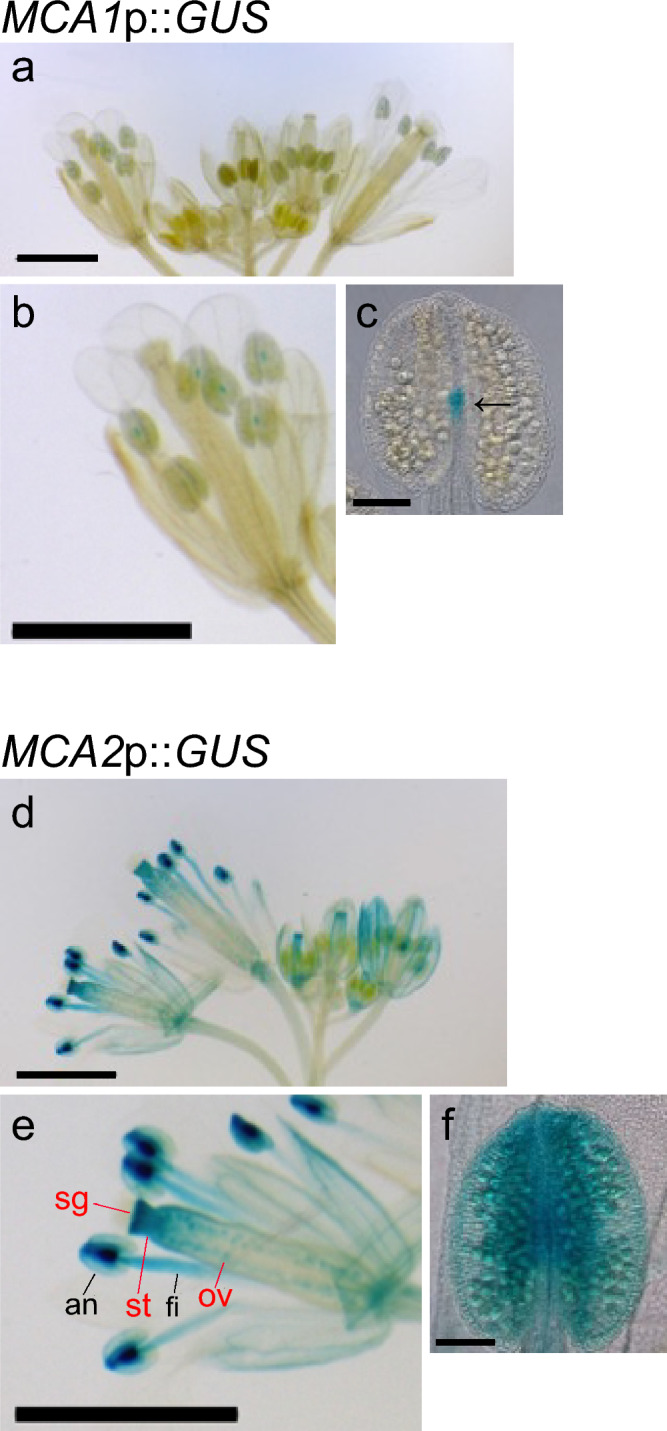
Tissue-specific expression patterns of *MCA1*p::*GUS* and *MCA2*::*GUS* in reproductive organs. GUS staining was performed on flowers at 45 DAS**. a** and **d**, flowers; **b** and **e**, enlarged photographs of one of the flowers shown in a and d, respectively. The stigma (sg), style (st), and ovule (ov) of the gynoecium were pointed out by red bars. The anther (an) and filament (fi) of the stamen were pointed out by black bars。The naming of tissues was based on Moubayidin and Østergaard (2017).; **c** and **f**, enlarged anther. The arrow in c indicates the position of the tip of the filament (Aloni et al. 2006). This figure shows a typical example from five experiments. Similar results were seen in the *MCA1*p::*GUS* lines 1, 3, 5, and 9, and the *MCA2*p::*GUS* lines 3, 4, and 5. Therefore, we examined at least 15 samples independently. Scale bar = 0.2 cm (**a**, **c**), 100 μm (**b**, **d**)

Reproductive organs are specialized structures with unique mechanosensory functions. Flower organs, such as stamen filaments and pistils, are sensitive to touch (Braam 2005). Although *MCA1*p::*GUS* expression was scarcely detected in reproductive organs (Fig. 8a, b), *MCA2*p::*GUS* was robustly expressed in various flower tissues. A closer look at the flowers under magnification before anthesis revealed the following interesting findings in relation to mechanical stress (Fig. 8b, e). The stamen consists of the anther and filament, both of which should receive mechanical forces during flower development due to contact with other organs and an active uptake of water (Ishiguro et al. 2001; Scott et al. 2004). Figure 8e shows that the intensity of GUS staining was high at the anther and the upper portion of the filament where water uptake is active (Ishiguro et al. 2001). Weak staining was observed in MCA1p::GUS flowers (Fig. 8b). In addition, the pistil also receives mechanical forces during flower development (Smyth 2005). It consists of the stigma, style, and ovary. Figure 8e shows that the intensity of GUS staining was high at the stigma and style, which supposedly encounter mechanical forces due to the elongation of the pistil during flower development (Bull–Hereñu et al. 2022). Thus, MCA2 may be involved in mechanosensing processes associated with pistil elongation.

## Spatiotemporal expression patterns in trichomes

**Fig. 9:**
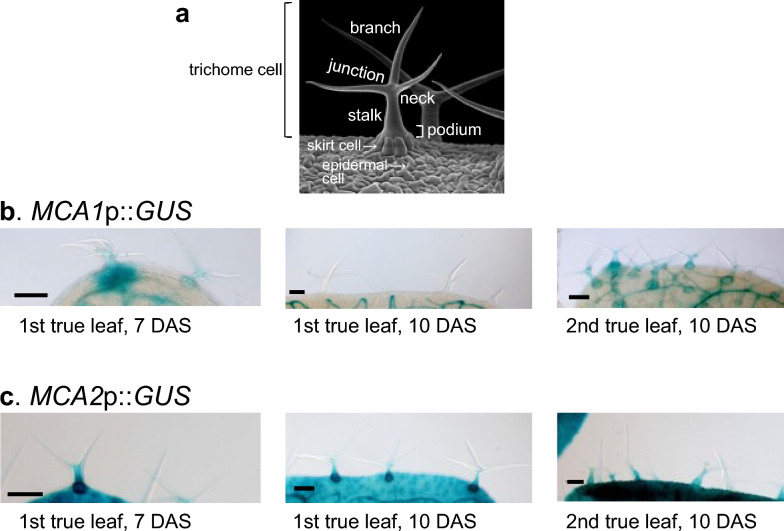
Expression patterns of *MCA1*p::*GUS* and *MCA2*::*GUS* in trichomes during true leaf development**. a.** Scanning electron micrograph of a trichome cell from *A. thaliana* with surrounding skirt cells and epidermal cells. The scanning electron micrograph taken by Stefan Eberhard was obtained online (https://wellcomecollection.org/works/pvacpmdu). A portion of the micrograph is presented here. Naming of the positions of a trichome cell and the surrounding cells followed Zhou et al. (2017) and Liu et al. (2017). GUS staining was performed on a set of the 1st true leaves of *MCA1*p::*GUS* (**b**) and *MCA2*p::*GUS* lines (**c**) plants at 7 DAS and that of the 2nd true leaves at 10 DAS. This figure shows a typical example from five experiments. Similar results were seen in the *MCA1*p::*GUS* lines 1, 3, 5, and 9, and the *MCA2*p::*GUS* lines 3, 4, and 5. Therefore, we examined at least 15 samples independently. Scale bar = 300 mm

Trichomes also exhibit specialized structures with unique mechanosensory functions (Fig. 9a). As shown in Fig. 9b and c, *MCA1*p::*GUS* and *MCA2*p::*GUS* were expressed in trichomes in a spatiotemporal manner with common and unique features, as both genes were strongly expressed in the skirt cells that support trichome cells; however, the expression of *MCA1*p::*GUS* became undetectable on the 1st true leaves at 10 DAS but was detectable at 7 DAS. In the newly formed true leaves, *MCA1*p::*GUS* was expressed even at 10 DAS. Therefore, age-dependent expression of *MCA1* may occur in both trichomes and skirt cells.

Trichomes are mechanosensory cells that are activated by mechanical stresses, such as brushing; help protect plants from herbivore attack; and participate in the plant immune response (Matsumura et al. 2022; Zhou et al. 2017). Zhou et al. (2017) showed that when trichomes were mechanically stimulated by compression or brushing, Ca^2+^ oscillations were induced in skirt cells. In addition, it is shown that when the neck of a trichome is flicked with a wire, Ca^2+^ influx is induced in the trichome base, and Ca^2+^ waves spread out to surrounding cells (Matsumura et al. 2017). Notably, these are the cells and locations at which *MCA1* and *MCA2* are highly expressed. Therefore, it is plausible that the two Ca^2+^-permeable mechanosensitive channels are involved in these physiological processes in trichomes and skirt cells.

## Conclusion

The dynamic expression patterns of MCA1 and MCA2 offer valuable insights into the roles of these proteins in plant growth and development. Based on their preferential expression in young tissues and specialized functions in reproductive organs and trichomes, MCA1 and MCA2 are likely key components that sense and respond to mechanical cues at an early stage of development. As the location and timing of the expression of *MCA1* and *MCA2* genes have been determined, we can focus on a specific organ at a limited timeframe and analyze it at the molecular level. Therefore, our knowledge of plant mechanosensing will undoubtedly be advanced by future studies aimed at dissecting the molecular mechanisms that underly the differential expression patterns of *MCA1* and *MCA2* genes and functional divergence. This knowledge should help researchers regulate plant growth under stress conditions and harness the potential of MCA1 and MCA2 in agricultural and biotechnological applications.

## Supplementary Information

Below is the link to the electronic supplementary material.

Supplementary file1 (PDF 127 KB)

## Data Availability

The original data used for this study are available upon request to the corresponding author.

## References

[CR1] Aloni R, Aloni E, Langhans M, Ulrich CI (2006) Role of auxin in regulatin Arabidopsis flower development. Planta 223:315–328 16208486 10.1007/s00425-005-0088-9

[CR2] Barton MK (2010) Twenty years on: The inner workings of the shoot apical meristem, a developmental dynamo. Dev Biol 341:95–113. 10.1016/j.ydbio.2009.11.029 19961843 10.1016/j.ydbio.2009.11.029

[CR3] Bull-Hereñu K, dos Santos P, Ginefra JF, El Ottra JHL, Thaowetsuwan P, Jeiter J, Ronse De Craene LP, Iwamoto A (2022) Mechanical forces in floral development. Plants 11:661. 10.3390/plants11050661 35270133 10.3390/plants11050661 PMC8912604

[CR4] Chiatante D, Montagnoli A, Trupiano D, Sferra G, Bryant J, Rost TL, Scippa GS (2021) Meristematic connectome: a cellular coordinator of plant responses to environmental signals? Cells 10:2544 34685524 10.3390/cells10102544 PMC8533771

[CR5] Dolan L, Janmaat K, Willemsen V, Linstead P, Poethig S, Roberts K, Scheres B (1993) Cellular organisation of the *Arabidopsis thaliana* root. Development 119:71–84. 10.1242/dev.119.1.71 8275865 10.1242/dev.119.1.71

[CR6] Furuichi T, Iida H, Sokabe M, Tatsumi H (2012) Expression of Arabidopsis MCA1 enhanced mechanosensitive channel activity in the *Xenopus laevis* oocyte plasma membrane. Plant Signal Behav 7:1022–1026 22751361 10.4161/psb.20783 PMC3474671

[CR7] Giorgio JAD, Bienert GP, Ayub ND, Yaneff A, Barberini ML, Mecchia MA, Amodeo G, Soto GC (2016) Muschietti, J.P. Pollen-specific aquaporins NIP4;1 and NIP4;2 are required for pollen development and pollination in *Arabidopsis thaliana*. Plant Cell 28:1053–1077 27095837 10.1105/tpc.15.00776 PMC4904668

[CR8] Guivarc’h A, Caissard JC, Azmi A, Elmayan T, Chriqui D, Tepfer M (1996) *In situ* detection of expression of the *gus* reporter gene in transgenic plants: ten years of blue genes. Transgenic Res 5:281–328 10.1007/BF01968938

[CR9] Hamant O, Heisler MG, Jönsson H, Krupinski P, Uyttewaal M, Bokov P, Corson F, Sahlin P, Boudaoud A, Meyerowitz EM, Couder Y, Traas J (2008) Developmental patterning by mechanical signals in Arabidopsis. Science 322(5908):1650–1655. 10.1126/science.1165594 19074340 10.1126/science.1165594

[CR10] Hamilton ES, Schlegel AM, Haswell ES (2015) United in diversity: Mechanosensitive ion channels in plants. Annu Rev Plant Biol 66:113–137 25494462 10.1146/annurev-arplant-043014-114700 PMC4470482

[CR11] Hattori T, Otomi Y, Nakajima Y, Soga K, Wakabayashi K, Iida H, Hoson T (2020) MCA1 and MCA2 are involved in the response to hypergravity in Arabidopsis hypocotyls. Plants 9:E590. 10.3390/plants9050590 10.3390/plants9050590 PMC7285502 32380659

[CR12] Heckwolf M, Pater D, Hanson DT, Kaldenhoff R (2011) The *Arabidopsis thaliana* aquaporin AtPIP1;2 is a physiologically relevant CO_2_ transport facilitator. Plant J 67:795–804 21564354 10.1111/j.1365-313X.2011.04634.x

[CR13] Hepler PK (2005) Calcium: a central regulator of plant growth and development. Plant Cell 17:2142–2155 16061961 10.1105/tpc.105.032508 PMC1182479

[CR14] Ishiguro S, Kawai-Oda A, Ueda J, Nishida I, Okada K (2001) The *DEFECTIVE IN ANTHER DEHISCENCE1* gene encodes a novel phospholipase A1 catalyzing the initial step of jasmonic acid biosynthesis, which synchronizes pollen maturation, anther dehiscence, and flower opening in Arabidopsis. Plant Cell 13:2191–2209 11595796 10.1105/tpc.010192 PMC139153

[CR15] Kurusu T, Nishikawa D, Yamazaki Y, Gotoh M, Nakano M, Hamada H, Yamanaka T, Iida K, Nakagawa Y, Saji H, Shinozaki K, Iida H, Kuchitsu K (2012) Plasma membrane protein OsMCA1 is involved in regulation of hypo-osmotic shock-induced Ca^2+^ influx and modulates generation of reactive oxygen species in cultured rice cells. BMC Plant Biol 12:11. 10.1186/1471-2229-12-11 22264357 10.1186/1471-2229-12-11 PMC3313898

[CR16] Kurusu T, Kuchitsu K, Nakano M, Nakayama Y, Iida H (2013) Plant mechanosensing and Ca^2+^ transport. Trends Plant Sci 18:227–233 23291244 10.1016/j.tplants.2012.12.002

[CR17] Liu S, Jiao J, Lu TJ, Xu F, Puckard BG (2017) Arabidopsis leaf trichomes as acoustic antennae. Biophy J 113:2068–2076. 10.1016/j.bpj.2017.07.035 10.1016/j.bpj.2017.07.035 PMC5685652 29117529

[CR18] Matsumura M, Nomoto M, Itaya T, Aratani Y, Iwamoto M, Matsuura T, Hayashi Y, Mori T, Skelly M, Yamamoto YY, Kinoshita T, Mori IC, Suzuki T, Betsuyaku S, Spoel SH, Toyora M, Tada Y (2022) Mechanosensory trichome cells evoke a mechanical stimuli–induced immune response in *Arabidopsis thaliana*. Nat Commun 13:1216. 10.1038/s41467-022-28813-8 35260555 10.1038/s41467-022-28813-8 PMC8904797

[CR19] Mori K, Na R, Naito M, Nakamura A, Shiba H, Yamamoto T, Suzaki T, Iida H, Miura K (2018) Ca^2+^-permeable mechanosensitive channels MCA1 and MCA2 mediate cold-induced cytosolic Ca^2+^ increase and cold tolerance in Arabidopsis. Sci Rep 8:550. 10.1038/s41598-017-17483-y 29323146 10.1038/s41598-017-17483-y PMC5765038

[CR20] Moubayidin L, Østergaard L (2017) Gynoecium formation: an intimate and complicated relationship. Curr Op Genet Dev 45:15–21. 10.1016/j.gde.2017.02.005 28242478 10.1016/j.gde.2017.02.005

[CR21] Murashige T, Skoog S (1962) A revised medium for rapid growth and bioassays with tobacco tissue cultures. Physiol Plant 15:473–497 10.1111/j.1399-3054.1962.tb08052.x

[CR22] Nakagawa Y, Katagiri T, Shinozaki K, Qi Z, Tatsumi H, Furuichi T, Kishigami A, Sokabe M, Kojima I, Sato S, Kato T, Tabata S, Iida K, Terashima A, Nakano M, Ikeda M, Yamanaka T, Iida H (2007) *Arabidopsis* plasma membrane protein crucial for Ca^2+^ influx and touch sensing in roots. Proc Natl Acad Sci USA 104:3639–3644 17360695 10.1073/pnas.0607703104 PMC1802001

[CR23] Nakano M, Iida K, Nyunoya H, Iida H (2011) Determination of structural regions important for Ca^2+^ uptake activity in Arabidopsis MCA1 and MCA2 expressed in yeast. Plant Cell Physiol 52:1915–1930 21949028 10.1093/pcp/pcr131

[CR24] Nakano M, Furuichi T, Sokabe M, Iida H, Tatsumi H (2021) The gravistimulation-induced very skow Ca^2+^ increase in Arabidopsis seedlings requires MCA1, a Ca^2+^-permeable mechanosensitive channel. Sci Rep 11:227. 10.1038/s41598-020-80733-z 33420331 10.1038/s41598-020-80733-z PMC7794229

[CR25] Nishii K, Möller M, Iida H (2021) Mix and match: Patchwork domain evolution of the land plant-specific Ca^2+^-permeable mechanosensitive channel MCA. PLoS ONE 16:e0249735 33857196 10.1371/journal.pone.0249735 PMC8049495

[CR26] Pond RH (1908) Emergence of lateral roots. Bot Gazette 46:410–421. 10.1086/329783 10.1086/329783

[CR27] Potocka I, Szymanowska-Pulka J, Jarcrewski J, Nakielski J (2011) Effect of mechanical stress on Zea root apex. I. Mechanical stress leads to the switch from closed to open merist4m organization. J Exp Bot 62:4583–4593 21659665 10.1093/jxb/err169 PMC3170553

[CR28] Rahni R, Birnbaum KD (2019) Week-long imaging of cell divisions in the Arabidopsis root meristem. Plant Methods 15:30. 10.1186/s13007-019-0417-9 30988691 10.1186/s13007-019-0417-9 PMC6446972

[CR29] Schres B, Benfey P, Dolan L (2002) Root development. Arabidopsis Book. 10.1199/tab.0101 10.1199/tab.0101 PMC3243376 22303222

[CR30] Scott RJ, Spielman M, Dickinson HQ (2004) Stamen structure and function. Plant Cell 16:S4–S60 10.1105/tpc.017012 PMC2643399 15131249

[CR31] Shigematsu H, Iid K, Nakano M, Chaudhuri P, Iida H, Nagayama K (2014) Structural Characterization of the Mechanosensitive channel candidate MCA2 from *Arabidopsis thaliana*. PLoS ONE 9:e87724. 10.0371/journal.pone.0087724 24475319 10.0371/journal.pone.0087724 PMC3903776

[CR32] Smyth DR (2005) Morphogenesis of flowers-our evolving view. Plant Cell 17:330–341 15689423 10.1105/tpc.104.030353 PMC548810

[CR33] Thor K (2019) Calcium-nutrient and messenger. Front. Plant Sci 10:440. 10.3389/fpls.2019.00440 10.3389/fpls.2019.00440 PMC6495005 31073302

[CR34] Toyota M, Spencer D, Sawai-Toyota S, Jiaqi W, Koo AJ, Howe GA, Giloy S (2018) Glutamate triggers long-distance, calcium-based plant defense signaling. Science 361:1112–1115 30213912 10.1126/science.aat7744

[CR35] Verbelen J-P, Cnodder TD, Le J, Vissenberg K, Baluška F (2006) The root apex of *Arabidopsis thaliana* consists of four distinct zones of growth activities: meristematic zone, transition zone, fast elongation zone and growth terminating zone. Plant Signal Behav 1:296–304. 10.4161/psb.1.6.3511 19517000 10.4161/psb.1.6.3511 PMC2634244

[CR36] Weig A, Deswarte C, Chrispeels MJ (1997) The Major intrinsic protein family of Arabidopsis has 23 members that form three distinct groups with functional aquaporins in each croup. Plant Physiol 114:1347–1357 10.1104/pp.114.4.1347 PMC158427 9276952

[CR37] Xu N, Cheng L, Kong Y, Chen G, Zhao L, Liu F (2024) Functional analyses of the NRT2 family of nitrate transporter in *Arabidopsis*. Front Plant Sci 15:1351998. 10.3389/fpls.2024.1351998 38501135 10.3389/fpls.2024.1351998 PMC10944928

[CR38] Yamanaka T, Nakagawa Y, Mori K, Nakano M, Imamura T, Kataoka H, Terashima A, Iida K, Kojima I, Katagiri T, Shinozaki K, Iida H (2010) MCA1 and MCA2 that mediate Ca^2+^ uptake have distinct and overlapping roles in *Arabidopsis thaliana*. Plant Physiol 152:1284–1296 20097794 10.1104/pp.109.147371 PMC2832256

[CR39] Yi H, Chen Y, Anderson CT (2022) Turgor pressure change in stomatal guard cells arises from interactions between water flux and mechanical responses of their cell walls. Quant Plant Biol 3(e12):1–8. 10.1017/qpb.2022.8 10.1017/qpb.2022.8 PMC10095868 37077969

[CR40] Yoshimura K, Iida K, Iida H (2021) MCAs in Arabidopsis are Ca^2+^-permeable mechanosensitive channels inherently sensitive to membrane tension. Nat Commun 12:6074. 10.1038/s41467-021-26363-z 34667173 10.1038/s41467-021-26363-z PMC8526687

[CR41] Zhou LH, Liu SB, Wang PF, Lu TJ, Xu F, Genin GM, Pickard BG (2017) The Arabidopsis trichome is a active mechanosensory switch. Plant Cell Environ 40:611–621 26920667 10.1111/pce.12728

