# Supplementary material for: Physiological roles of Arabidopsis MCA1 and MCA2 based on their dynamic expression patterns
Source: J Plant Res. 2024 Aug 28;137(5):785–97. doi: 10.1007/s10265-024-01575-8 (PMC11393015; doi:10.1007/s10265-024-01575-8)
Supplement: Supplementary file 1 — Supplementary file1 (PDF 127 KB) [file 10265_2024_1575_MOESM1_ESM.pdf]

Electronic supplementary materials

Title:

Physiological roles of Arabidopsis MCA1 and MCA2 based on their dynamic expression patterns

Authors:

Miki Kubota[1,2], Kendo Mori[1,3], and Hidetoshi Iida[1] *

[1]*Department of Biology, Tokyo Gakugei University, 4-1-1 Nukuikita-machi, Koganei, Tokyo 184-8501, Japan*

[2]Present address: *Kawagoe Minami High School, 1-21-1 Minamiotsuka, Kawagoe, Saitama 350-1162, Japan*

[3]Present address: *Tamagawa Academy High School, 2713 Naracho, Aoba Ward, Yokohama, Kanagawa 227-0036, Japan*

Journal:

Journal of Plant Research

***Corresponding author:**

Hidetoshi Iida

Department of Biology, Tokyo Gakugei University, Koganei, Tokyo 184-8501, Japan

iida@u-gakugei.ac.jp

Content:

Materials and methods

Table S1

Materials and methods

Plant materials and growth conditions

The Columbia-0 (Col-0) ecotype of *Arabidopsis thaliana* (Arabidopsis) and its isogenic, transgenic lines were used in this study. The *MCA1* and *MCA2* promoters fused to the reporter gene β-glucuronidase (GUS), *MCA1*p::*GUS* and *MCA2*p::*GUS*, respectively, have been described previously (Yamanaka et al., 2010) and were used to localize the expression of MCA1 and MCA2. The *MCA1*p::*GUS* lines 1, 3, and 5 and the *MCA2*p::*GUS* lines 3, 4, and 5 were obtained from our laboratory's stock.

Seeds were treated for 10 min with 1 mL of 5% sodium hypochlorite/10% Triton X-100 solution, washed five times with autoclaved Milli-Q water (1 mL each), and stored for 3 days at 4 °C in the dark. Then, the seeds were sown on Murashige and Skoog (MS) media (Murashige and Skoog, 1962; FUJIFILM Wako Pure Chemical Corp., Osaka, Japan) supplemented with 0.8% (w/v) agar (Difco Laboratories, Detroit, Michigan, USA), and the germinated seedlings were grown under a 16 h light/8 h dark cycle with white light from fluorescent lamps at approximately 90-110 μmol m^{-2} s^{-1} light intensity at 22 °C.

Histochemical analysis

The plants were placed in 1.5 mL Eppendorf tubes containing 1 mL of 90% acetone and placed on ice for 1 h for fixation. The plants were then washed twice with 1 mL of 0.1 M sodium phosphate buffer (5x dilution of 0.5 M sodium phosphate buffer, pH 7.0). Then, 1 mL of X-Gluc buffer (0.1 M sodium phosphate buffer (pH 7.0), 0.5 mg/ml X-Gluc [5-bromo-4-chloro-3-indolyl glucuronidase; Bio Medical Science, Ltd., Tokyo], 0.5 mM potassium ferrocyanide, and 0.5 mM potassium ferricyanide) was added, and the mixture was incubated at 37 °C in an incubator (Sanyo, MLR-153) for 12-18 h. The plants were then decolorized (50% → 70% → 90% → 100%) by immersion in ethanol (09-0770-3; Sigma–Aldrich Japan) at different concentrations in stages. The samples were soaked in 50% ethanol for 10 min and sterile Milli-Q water for 10 min. Then, the

samples were soaked in sterilized Milli-Q water for preparation and observed and photographed using a binocular stereomicroscope (Leica, DFC300FX, Germany) or phase contrast microscope (Olympus, 3G05308, Tokyo, Japan).

Semiquantitative RT–PCR

Total RNA was obtained from the whole organs of interest within Arabidopsis plants at 10, 15, and 20 days after sowing (DAS) with a NucleoSpin RNA Plant Kit (Macherey-Nagel, Rev. 06.1012/001; Allentown, PA, USA) according to the manufacturer's protocol. cDNA was synthesized for 30 min at 37 °C with oligo (dT) primers and a PrimeScript RT reagent kit (RR037A; Takara, Shiga, Japan) on a T00 Thermal Cycler (186-1096J1; Bio-Rad, Hercules, CA, USA). RT–PCR was performed using the primer pairs listed in Table S1 on a T00 Thermal Cycler. Each reaction was carried out in a mixture (9 µl) containing 1 µl of 40 ng/µl cDNA, 5 µl of 2x Quick Taq HS DyeMix (Toyobo, Osaka, Japan), 0.2 µl of 10 µM forward primer, 0.2 µl of 10 µM reverse primer, and 3.6 µl of Milli-Q water. The thermal profile consisted of 30 s of hot-start enzyme activation at 94 °C, followed by 32 cycles of PCR at 94 °C for 2 min (denaturation), 55 °C for 30 s (annealing), and 68 °C for 1 min (elongation). After the reaction, 5 µl of the reaction mixture and 1 µl of 6x GR Red loading buffer (Bio Craft, Tokyo, Japan) were mixed, and 5 µl of the mixture was electrophoresed for 20 min at 100 V on a 2% agarose gel. Following electrophoresis, the gels were photographed with a ChemiDoc XRS+ (Bio-Rad), and the fluorescence intensity of each PCR product in the gel was quantified with the image processing software ImageJ. The intensity of the *MCA1* and *MCA2* products is indicated by the relative value when that of β-tubulin was set to 1.

References

Murashige T, Skoog S (1962) A revised medium for rapid growth and bioassays with tobacco tissue cultures. *Physiol. Plant.* 15, 473–497.

Table S1 List of primers used for RT–PCR

Name of primer	Nucleotide sequence (5’ to 3’)
FAM1AG0007 (*MCA1*-f[1])	TGCTCCCTCTTTTAGTCAATTCTC
RAM1Ao981 (*MCA1*-r[2])	CCTGTTGGATGCTGCAGTGGCAATC
FAM1B565S1 (*MCA2*-f)	CATCCGTAATTTGCTAGAGCA
FAM1BG0129 (*MCA2*-r)	ACAAGTACCATCTCTGTAATTCTTGAC
FAM1B630S2 (*MCA2*-f)	CGCTTCAAGGACTTGATGATGATGC
RAM1Bo888 (*MCA2*-r)	CAAGGTTCTGAACAACAATCCAGC
Tubulin-f	ACCACTCCTAGCTTTGGTGATCTG
Tubulin-r	AGGTTCACTGCGAGCTTCCTCA

[1]f, forward; [2]r, reverse.
